# Directly synthesized p-type PbS quantum-dot inks enable uniform SWIR imaging

**DOI:** 10.1093/nsr/nwag541

**Published:** 2026-09-07

**Authors:** Yilong Zhou, Zhixian Zhang, Jiang Tang

**Affiliations:** Wuhan National Laboratory for Optoelectronics (WNLO) and School of Optical and Electronic Information (SOEI), Huazhong University of Science and Technology, China; JFS Laboratory, China; Wuhan National Laboratory for Optoelectronics (WNLO) and School of Optical and Electronic Information (SOEI), Huazhong University of Science and Technology, China; Wuhan National Laboratory for Optoelectronics (WNLO) and School of Optical and Electronic Information (SOEI), Huazhong University of Science and Technology, China; Optics Valley Laboratory, China

Short-wave infrared (SWIR) imaging unlocks structural, chemical and environmental information beyond the visible spectrum. Conventional epitaxial semiconductors for SWIR detection, however, suffer from high manufacturing costs and complex integration processes, which hinder their widespread deployment. Solution-processed lead sulfide (PbS) quantum dots (QDs) offer a promising alternative: their bandgap is widely tunable across the infrared range, and they can be deposited directly onto silicon readout integrated circuits [1,2]. Translating nanoscale surface-chemistry control into uniform performance across hundreds of thousands of pixels at wafer scale, nevertheless, remains a long-standing manufacturing challenge.

This challenge is especially acute in PbS QD p–n homojunction devices. While conductive n-type QD inks are well established, high-quality p-type layers have long relied on solid-state ligand exchange (SSLE) performed after film deposition. Post-deposition ligand replacement enables charge transport, but incomplete ligand replacement, volumetric contraction and non-uniform oxidation can generate film cracks and disordered energetic landscapes [3]. Variations tolerable in individual photodiodes translate into photoresponse non-uniformity and defective pixels in large-area imaging arrays.

In a recent work published in *National Science Review*, Liu *et al*. overcame this bottleneck by developing directly synthesized p-type PbS QD inks, which encode electronic functionality during QD growth rather than via post-deposition conversion [4]. As shown in Fig. 1B, the synthesis uses lead thiocyanate [Pb(SCN)₂] as the lead precursor and para-substituted thiols as *in situ* surface ligands, encoding p-type electronic character during QD formation. The electron-withdrawing strength of the thiol substituents can be tuned to modulate ligand binding, QD growth kinetics and Fermi-level position. Combined with a seeded growth strategy, the QD diameter can be tuned from ∼3.2 to 8.2 nm, shifting the first-excitonic absorption peak from ∼1100 nm to beyond 2000 nm. This establishes a versatile p-type ink platform in which optical absorption and electronic properties can be engineered together at the synthesis stage.

**Figure 1.**
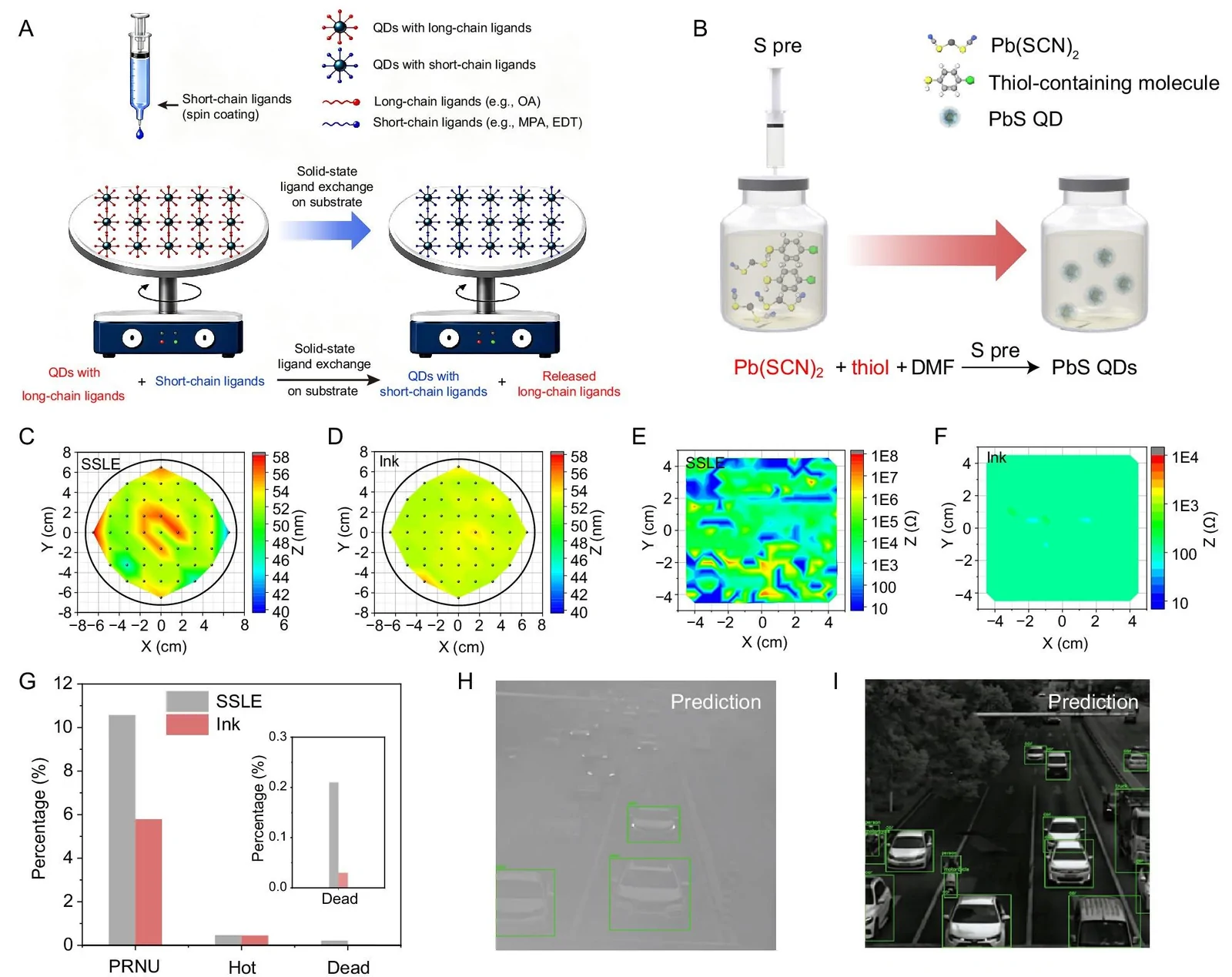
Direct p-type PbS quantum-dot inks translate synthesis-level control into uniform SWIR imaging. (A) Schematic of conventional SSLE, where long native ligands in deposited PbS QD films are replaced by short ligands. (B) Schematic of direct p-type PbS QD ink synthesis using Pb(SCN)₂ and thiol ligands. DMF: *N,N*-dimethylformamide. (C and D) Ellipsometric thickness maps of SSLE-derived and ink-derived films on 6-inch silicon substrates. (E and F) Sheet-resistance maps of SSLE and ink-based conductive films. (G) Comparison of photoresponse non-uniformity, hot-pixel fraction and dead-pixel fraction between SSLE and ink-based imagers. (H and I) Representative visible and SWIR images acquired under foggy conditions, with annotated boxes marking target objects. Panel A was drawn by the authors; panels B–I were adapted from Ref. [4].

The advantage of synthesis-level control becomes striking at wafer scale. Ink-derived QD films are dense and crack-free, with the full width at half maximum of the surface-potential distribution narrowing from 83 to 8 mV. On 6-inch silicon substrates, thickness variation drops from ±7.82 to ±0.31 nm, and sheet-resistance mapping shows markedly improved electrical uniformity (Fig. 1C–F). Fully ink-processed p–n photodiodes deliver single-pixel performance comparable to SSLE controls, along with lower noise, higher specific detectivity and enhanced operational stability.

Monolithic integration of the QD photodiodes with a 640 × 512 complementary metal-oxide-semiconductor (CMOS) readout circuit directly translates materials uniformity into imaging quality. Photoresponse non-uniformity decreases from 10.57% to 5.79%, and the dead-pixel fraction falls from 0.21% to 0.03% (Fig. 1G). The imager enables silicon-penetrating and chemical-contrast imaging and preserves substantially more scene information than visible imaging under foggy conditions, enabling more reliable vehicle recognition (Fig. 1H and I).

Together, these achievements establish directly synthesized p-type QD inks as a powerful materials platform for scalable, high-performance SWIR optoelectronics. By carrying molecularly defined electronic functionality from synthesis through wafer-scale processing and ultimately into pixel-consistent infrared vision, this work provides a promising route toward scalable large-area SWIR imaging systems.

## References

[1] Liu J, Liu P, Chen D et al. Nat Electron 2022; 5: 443–51. 10.1038/s41928-022-00779-x

[2] Liu M, Yazdani N, Yarema M et al. Nat Electron 2021; 4: 548–58. 10.1038/s41928-021-00632-7

[3] Liu M, Voznyy O, Sabatini R et al. Nat Mater 2017; 16: 258–63. 10.1038/nmat4800 27842072

[4] Liu Y, Ma S, Deng C et al. Natl Sci Rev 2026; 13: nwag421. 10.1093/nsr/nwag421 PMC13525715 42668928

